# Novel inhalation therapy in pulmonary fibrosis: principles, applications and prospects

**DOI:** 10.1186/s12951-024-02407-6

**Published:** 2024-03-29

**Authors:** Meiling Zheng, Wei Zhu, Fei Gao, Yu Zhuo, Mo Zheng, Guanghao Wu, Cuiling Feng

**Affiliations:** 1 Present Address: Dongzhimen Hospital, Beijing University of Chinese Medicine, Beijing, 100010 China; 2 Peking University People’s Hospital, Beijing, 100032 China; 3 School of Medical Technology, Beijing Institute of Technology, Beijing, 100081 China; 4 Department of Ophthalmology, Changshu No. 2 People’s Hospital, Changshu, 215500 China; 5 State Key Laboratory of Southwestern Chinese Medicine Resources, Pharmacy School, Chengdu University of Traditional Chinese Medicine, Chengdu, 611130 China; 6 Department of Medical Oncology Beijing Tsinghua Changgung Hospital, School of Clinical Medicine, Tsinghua University, Beijing, 100010 China

**Keywords:** Inhalation therapy, Pulmonary fibrosis, Nebulization, Dry powder, Nanomedicine

## Abstract

Pulmonary fibrosis (PF) threatens millions of people worldwide with its irreversible progression. Although the underlying pathogenesis of PF is not fully understood, there is evidence to suggest that the disease can be blocked at various stages. Inhalation therapy has been applied for lung diseases such as asthma and chronic obstructive pulmonary disease, and its application for treating PF is currently under consideration. New techniques in inhalation therapy, such as the application of microparticles and nanoparticles, traditional Chinese medicine monomers, gene therapy, inhibitors, or agonists of signaling pathways, extracellular vesicle interventions, and other specific drugs, are effective in treating PF. However, the safety and effectiveness of these therapeutic techniques are influenced by the properties of inhaled particles, biological and pathological barriers, and the type of inhalation device used. This review provides a comprehensive overview of the pharmacological, pharmaceutical, technical, preclinical, and clinical experimental aspects of novel inhalation therapy for treating PF and focus on therapeutic methods that significantly improve existing technologies or expand the range of drugs that can be administered via inhalation. Although inhalation therapy for PF has some limitations, the advantages are significant, and further research and innovation about new inhalation techniques and drugs are encouraged.

**Graphical Abstract:**

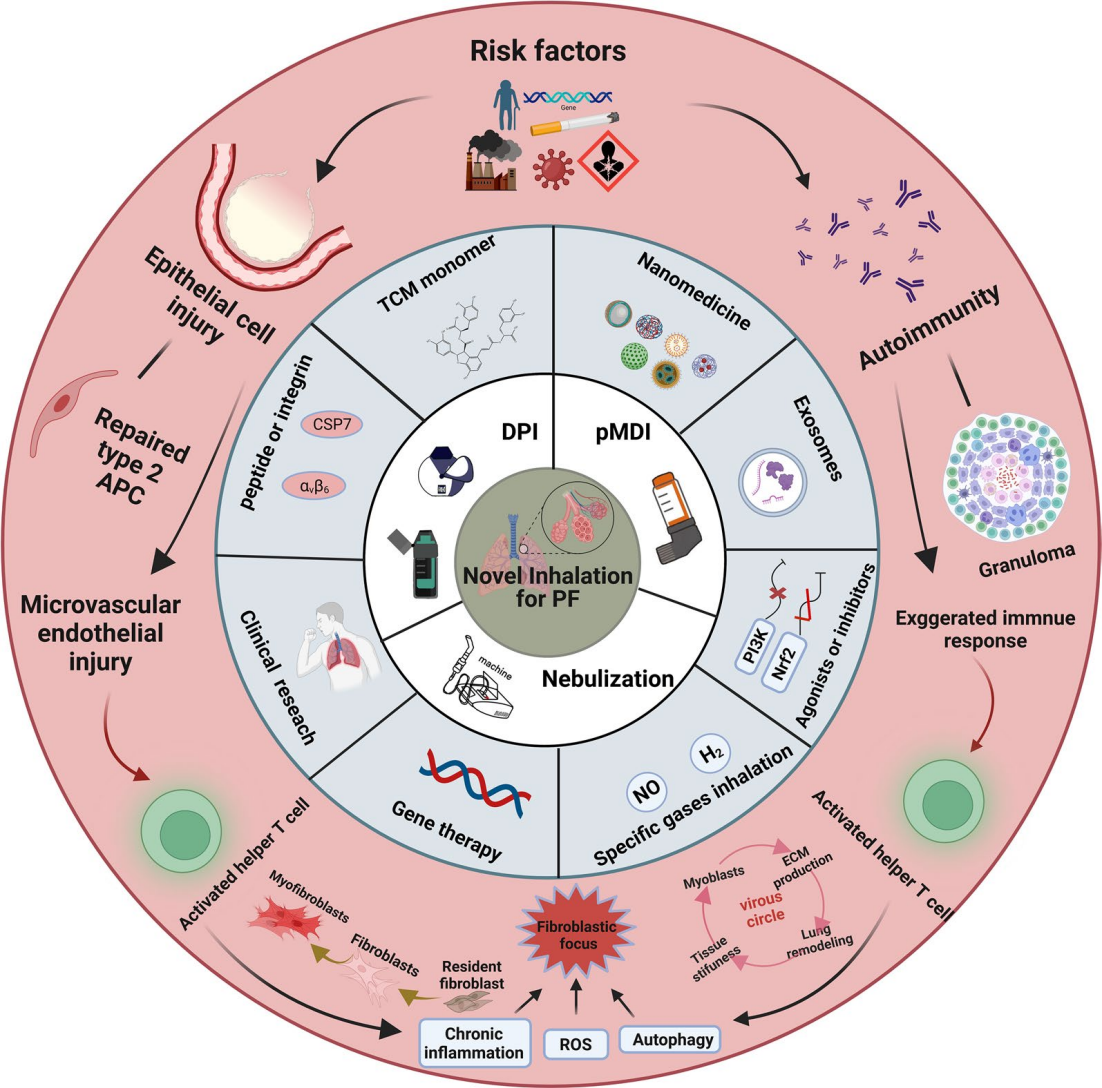

## Introduction

Pulmonary fibrosis (PF) can seriously threaten human health and quality of life. The incidence of idiopathic pulmonary fibrosis (IPF) has increased considerably, ranging from 8 to 60 cases per 100,000 individuals [1–3]. Pneumoconiosis and cystic pulmonary fibrosis (CPF) also affect hundreds of thousands of people worldwide [4, 5]. PF is a common characteristic of various diseases, including fibrous interstitial lung disease (ILD), pneumoconiosis, and CPF. IPF contributes to most of the ILD [5–7]. Multiple lung conditions, such as chronic obstructive pulmonary disease (COPD), acute/chronic lung injury, and radiation-induced and immune checkpoint inhibitor-related lung injury, can lead to PF [8, 9].

Chronic cough, sputum, and progressive dyspnea are common clinical symptoms of PF. For phenotypes that manifest as progressive pulmonary fibrosis (PPF), providing effective and safe treatment options that can help prolong life expectancy is necessary [6]. The official clinical practice guideline developed by the American Thoracic Society (ATS), European Respiratory Society (ERS), Japanese Respiratory Society (JRS), and Latin American Thoracic Association (ALAT) recommended pirfenidone (PFD) and Nintedanib as oral administrations for the treatment of mild-to-moderate IPF [6]. Although the exact mechanism of action by which PFD treats IPF is yet to be fully understood, studies have shown that both drugs are effective in preventing a decline in forced vital capacity (FVC) [10–12]. However, evidence on whether the two drugs can inhibit acute exacerbations of IPF, reduce mortality, or be applied to non-IPF PPF is insufficient. Moreover, the high costs limit their wide availability for clinical application. In addition to medication, guidelines recommend that oxygen supplementation and pulmonary rehabilitation as non-pharmacological therapies. Lung transplantation is a feasible treatment notion for patients with IPF, but absolute and relative contradictions to lung transplantation [13], combined with a severe shortage of donors, restrict its further implementation. PF is a pathological process that involves the damage and repair of lung tissue, characterized by chronic inflammation, oxidative stress, immune activation, and fatty acid metabolism [14–17]. Fibroblast synthesis and extracellular matrix (ECM) deposition are protective repair responses to tissue injury, while PF is a heterogeneous condition and may progress irreversibly in some cases. The underlying pathological process of PF involves various factors, including genetic and epigenetic modulation, infections, smoking, exposure to occupational/environmental pollutants, chemoradiotherapy, reflux/microaspiration, and the use of specific drugs such as bleomycin (BLM) and amiodarone [18].

**Fig. 1:**
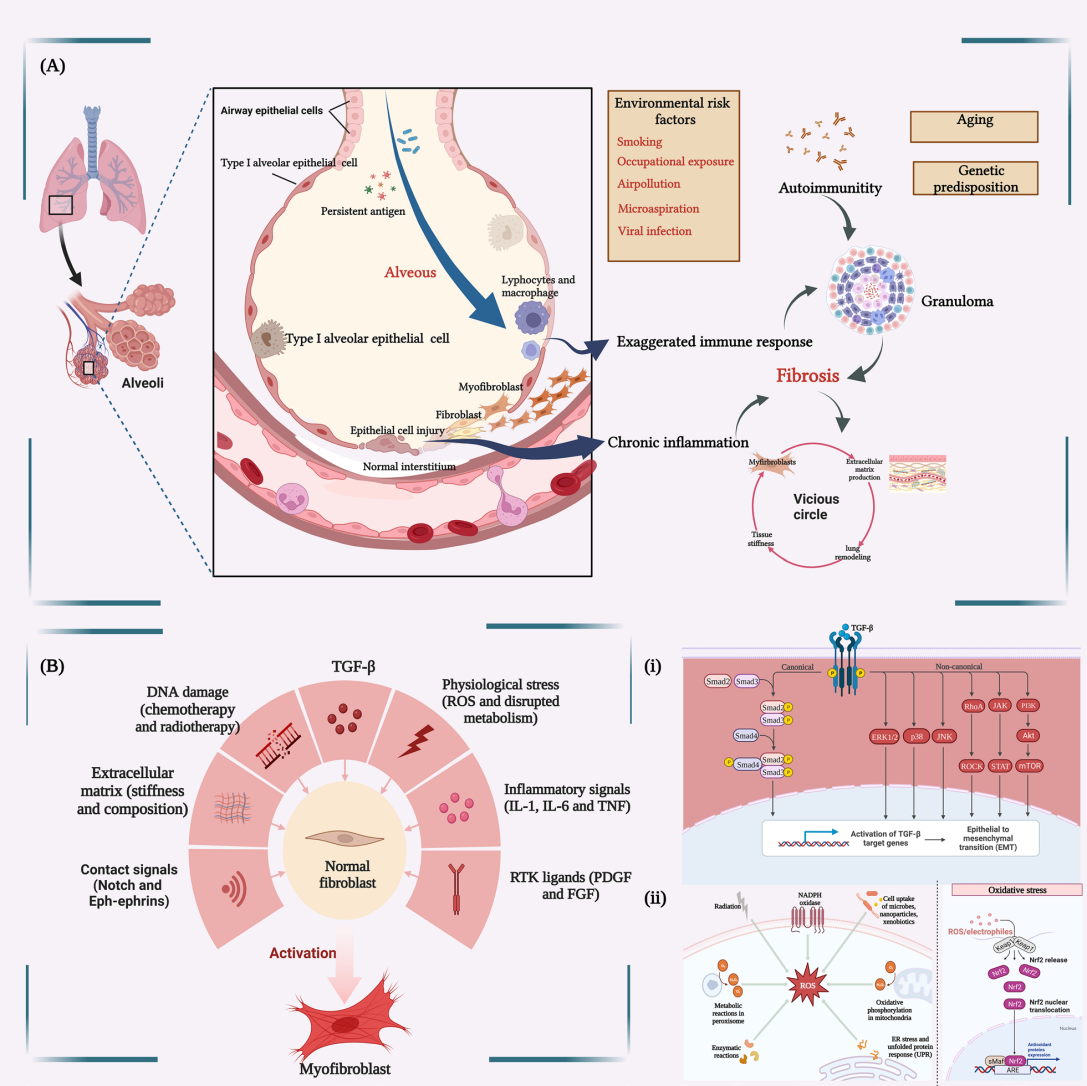
Main pathological and physiological mechanisms of pulmonary fibrosis. **A** The pathological process of occurrence and development of pulmonary fibrosis. **B** The associated pathological mechanisms of pulmonary fibrosis, such as oxidative stress, inflammatory response, DNA damage, and extracellular matrix deposition. **i** The TGF-β/SMAD signaling pathway involved in the progression of pulmonary fibrosis. **ii** The signaling pathways associated with oxidative stress in pulmonary fibrosis. This figure is created by BioRender and has confirmed publishing and licensing rights, with agreement number MQ256A249R

The transformation of cellular phenotypes and activation of signaling pathways might participate in the pathological process [19]. Epithelial dysfunction is a key component of the pathophysiological process of PF, and factors that trigger epithelial dysfunction induce damage to alveolar epithelial cells (AECs), which can lead to pathological alveolar epithelial remodeling. Alveolar type II epithelial cells (AT2) are the major epithelial progenitor cells that secrete surface-active protein C (SP-C) and play a key role in maintaining alveolar homeostasis [20, 21]. A study conducted single-cell sequencing of epithelial cells from IPF patients and found high enrichment of pathological epithelial cell populations, which can promote the deposition of ECM. The findings reaffirmed the perspective of repetitive epithelial cell damage [22]. The deposition of ECM, induced by the activation of fibroblasts and myofibroblasts, is the predominant stage of PF [23]. When multiple environmental triggers interact with genetic phenotypes, disordered or excessive ECM deposition may occur in the lung [24]. Fibroblasts and the deposition of ECM propagate fibrosis via a positive feedback loop, which leads to lung remodeling, tissue stiffness, and dyspnea [25, 26], as portrayed in Fig. 1A.

The pathogenesis of IPF involves multiple pathological processes, including DNA damage, transforming growth factor beta (TGF-β) signaling transduction, oxidative stress, inflammatory stimulation, and ECM deposition [18]. These mechanisms interact with each other and collectively contribute to the development and progression of IPF, as portrayed in Fig. 1B. Along with the TGF-β signaling pathway, the development of PF involves several other molecular signaling pathways. TGF-β is a pro-fibrotic factor that stimulates ECM production by fibroblasts, and downstream effectors of TGF-β1, such as phosphorylated SMAD 2/3 proteins, regulate target gene expression and contribute to fibrosis [27–29]. The TGF-β system stimulates the hedgehog signaling pathway and the integrin alpha-v beta-6 (avβ6) protein to promote PF [30, 31]. Additionally, oxidative stress and inflammation-related signaling pathways are also implicated in the pathogenesis of PF [17]. Reactive oxygen species (ROS) are produced intracellularly, mainly through the members of the nicotinamide adenine dinucleotide phosphate (NADPH) oxidase family, such as NADPH oxidase 4 (NOX4), and are induced in the mitochondria by the binding of TGF-β to receptors. This binding enhances TGF-β gene expression via various signaling pathways, such as nuclear factor erythroid 2-related factor 2/Heme oxygenase-1 (Nrf2/HO-1), Janus kinase 2/signal transducer and activator of transcription 3 (JAK2/STAT3), and p38 Mitogen-activated protein kinase/Nuclear factor kappa-light-chain-enhancer of activated B cells (p38 MAPK/NF-κB), and contributes to collagen deposition, which aggravates the progression of PF [32]. Multiple pro-fibrotic molecular mechanisms exist among cells, which highlights the complexity of the pathogenesis of PF, as portrayed in Fig. 1(i) and (ii).

New treatment methods, particularly novel inhalation therapy, have emerged. Inhalation therapy has significant advantages in COPD and asthma, and thus, it might be used for treating PF. With the advancement of nanoplatform technology, inhalation therapy has improved considerably, dominated by the enhancement of microparticles (MPs)/nanoparticles (NPs) technologies, including drug nano-size systems and nano-carrier drug delivery systems. Inhibitors or agonists of relevant signaling pathways, exosomes, and other novel specific drugs also benefit through the advancement of inhalation therapy.

Overall, although significant progress has been made in the understanding of PF, the clinical management of PF remains unsatisfactory. However, the emergence of novel inhalation therapeutic techniques presents new opportunities for the treatment of PF, and they also bring certain challenges that require further attention. In this review, we provided an overview of the benefits and challenges of inhalation therapy, the categories of inhalation therapy, and the application of various methods of novel inhalation therapy for treating PF. This review might contribute to the advancement of the field and improvement of the clinical management of PF.

## Classification of inhalation therapy

Inhalation therapy is essential for converting the target drug into aerosols, and various devices have been developed to achieve this, including nebulizer, pressure metered-dose inhaler (pMDI), dry powder inhaler (DPI), and soft mist inhaler [33]. These devices have certain advantages and disadvantages, and they are suitable for different therapeutic applications, as detailed in Table 1.

**Table 1:** The advantages and challenges of different inhalation devices

Inhalation devices	Specifical class	Personalized advantage	Personalized challenges	Overall advantage	overall challenges
Nebulizers [34–41]	Air-jet nebulizer	Easy maintenance and convenient operation	Unable to achieve lung deposition through downward spraying	Delivering high doses of medication Suitable for patients of multiple age groups No propellant needed Suitable for drugs that cannot be made into powder	Require a large quantity, easily lead to medication waste Improper cleaning of the reusable machine can result in microbial residue Not conducive to portability
	Ultrasonic nebulizer	High fog output	Machine heating may affect drug performance		
	Mesh nebulizer	Less noisy Higher lung deposition rates Lower agent residuals High stability	Not suitable for long-term operation		
	VMN	Delivering homogeneous-sized aerosol particles (1–5 µm) Higher lung deposition rates	More noise More expensive		
Dry powder inhalation [42–46, 48]	DPI	No outside energy sources	Challenging for those with impaired lung function and poor hand-mouth coordination Sensitivity to inhalation flow rate	Portable and compact Reduced medication dosage requirement Longer shelf life No priming required	High oropharyngeal deposition rate Limitations based on medication properties
	pMDI	Good for inhaled corticosteroids Useful for patients who cannot coordinate inhalation and actuation	Propellant dependency Limited dose counters		
Soft mist inhalers [158]	NA	Ease of inhalation High nebulization efficiency and lung deposition No coordination required Adjustable dosing	High cost Limitations based on medication properties Requires proper technique	NA	NA

### Nebulization

Nebulization is the conversion of drugs dissolved in liquids (solution or suspension) into a fine mist that can be inhaled through nebulizers. It has a long history of medical applications. Nebulizers are suitable for patients who have difficulty using other types of inhalers, such as children, elderly patients, or those with severe respiratory conditions. The air-jet nebulizer, ultrasonic nebulizer, mesh nebulizer, and vibrating mesh nebulizer (VMN) are presented in the order in which they were launched in the market.

Several factors contribute to the properties of the aerosols produced by nebulization. When selecting a nebulization device for inhalation therapy, one must consider various factors, even when dealing with the same drug formulations. For instance, air-jet nebulization leads to a decrease in solution temperature (24 °C to 17 °C) due to solvent evaporation, resulting in the production of very small droplets [34]. Ultrasonic nebulization, on the other hand, initially increases the liquid temperature due to ultrasonic vibration transfer, which reduces drug concentration and viscosity, and lowers surface tension, leading to the formation of small droplets. However, the droplet size distribution significantly depends on nebulization time, with air-jet nebulization showing an increase in droplet size in the first 2 min before decreasing, while ultrasonic nebulization experiences a decrease in droplet size in the first 4 min, followed by an increase for the next 2 min and then a decrease in the last 4 min. Moreover, temperature changes can affect surface tension, concentration, viscosity, and particle size of aerosols. Therefore, the drug formulation and the characteristics of the nebulizer must be thoroughly considered when selecting the optimal device for inhalation therapy.

Mesh nebulizers represent a recent technological advancement in nebulizers that combine the advantages of compression and ultrasonic nebulizers. They use ultrasonic vibrations and mesh aerosol nozzle configuration to spray liquid through the orifices of the nozzle-type mesh aerosol nozzle. These nebulizers offer numerous benefits, such as being less noisy, more portable, and having higher lung deposition rates, lower agent residuals, and high stability [35]. Lin et al. showed that the performance of VMN in delivering salbutamol was not affected, although it was used without cleaning for more than 28 days, which indicated the stability of VMN [36]. The VMN consistently delivers homogeneous-sized aerosol particles (1–5 µm) to ensure optimal lung deposition rates and individual single use to avoid cross-infection. Studies have shown that mesh nebulizers are particularly effective for delivering inhaled antibiotics in the treatment of CPF [37]. However, VMN is susceptible to the physicochemical properties of the liquid and the atomization operating mechanism, particularly with more viscous liquids [38]. Therefore, when selecting the optimal nebulizer for inhaled antibiotics, the physicochemical properties of the liquid and the atomization operating mechanism should also be considered.

Nebulizers have some limitations that should be considered when selecting the optimal device for inhalation therapy. One limitation is the wastage of drugs caused by nebulizers [39]. Nebulizers require considerably larger therapeutic doses than pMDI and DPI, which can cause substantial drug loss; VMN has the least loss of drugs, and air-jet nebulizer has the most. For example, the nebulized dose of salbutamol sulfate solution is 5 mg/dose, whereas the dose of salbutamol sulfate aerosol is around 200 µg/dose for long-term usage. Additionally, home nebulizers following repeated usage without adequate cleaning and disinfection might cause surface bacteria to disperse, which might have an adverse effect. Harris et al. inoculated bacteria isolated from CPF patients into nebulizers donated by CPF patients and collected aerosols [40]. Fluorescence microscopy was performed to observe the dispersion of bacteria during the re-nebulization of salbutamol, especially under high humidity. However, many patients and their families are unaware of the importance of cleaning and disinfecting nebulizers or do not disinfect them thoroughly [41]. Therefore, nebulizers with good performance should consider effectiveness, safety, portability, stability, and low cost, among other factors. Patients and their families need to be informed about the proper use, cleaning, and disinfection of nebulizers to ensure optimal treatment outcomes.

### Pressurized metered-dose inhaler and dry powder inhaler

pMDI have been used for decades. In these devices, an external pressure pushes the valve to pump the solution or suspension out of the closed container. These pMDI devices shifted from using chlorofluorocarbons (CFC) to hydrofluoroalkanes, which improved their performance, for example, reducing the spray velocity and generating smaller aerosols for greater lung deposition [42].

DPI was developed in the 1980s and widely applied for inhalation therapy. DPI devices can be classified as single-dose capsules, multi-dose reservoirs, and vesicles. They depolymerize drugs based on the inspiratory flow rate, the peak inspiratory flow rate (PIFR) of patients, and the resistance built into the device [43, 44]. PIFR was found to be significantly associated with the depolymerization and lung deposition rate of the drugs [45]. DPI needs patients to start the initial deep and vigorous breathing, which might be challenging for those with impaired lung function and poor hand-mouth coordination. However, some studies have shown that patients with diseases such as COPD, asthma, and CPF can generate the inspiratory flow rate required for DPI [46, 47].

Both pMDI and DPI have common problems, such as the dose of drugs delivered, and the pulmonary deposition rates are low. The pulmonary deposition rate is about 20–40% for both, and up to 48% for pMDI with co-suspension technology [48], which implies that more than half of the drugs do not reach the target site. Patient adherence and inhalation techniques are also essential for sustaining therapeutic effects, and regular follow-ups and assessment of the inhalation performance of the patient are necessary. Overall, pMDI and DPI are effective options for the inhalation therapy of COPD, asthma, and other respiratory diseases. However, the limitations and challenges associated with each device need to be considered when selecting the optimal device for each patient. Additionally, patient education and regular follow-ups are necessary to ensure optimal treatment outcomes.

## Advantages and challenges of inhalation therapy

### Advantages

**Fig. 2:**
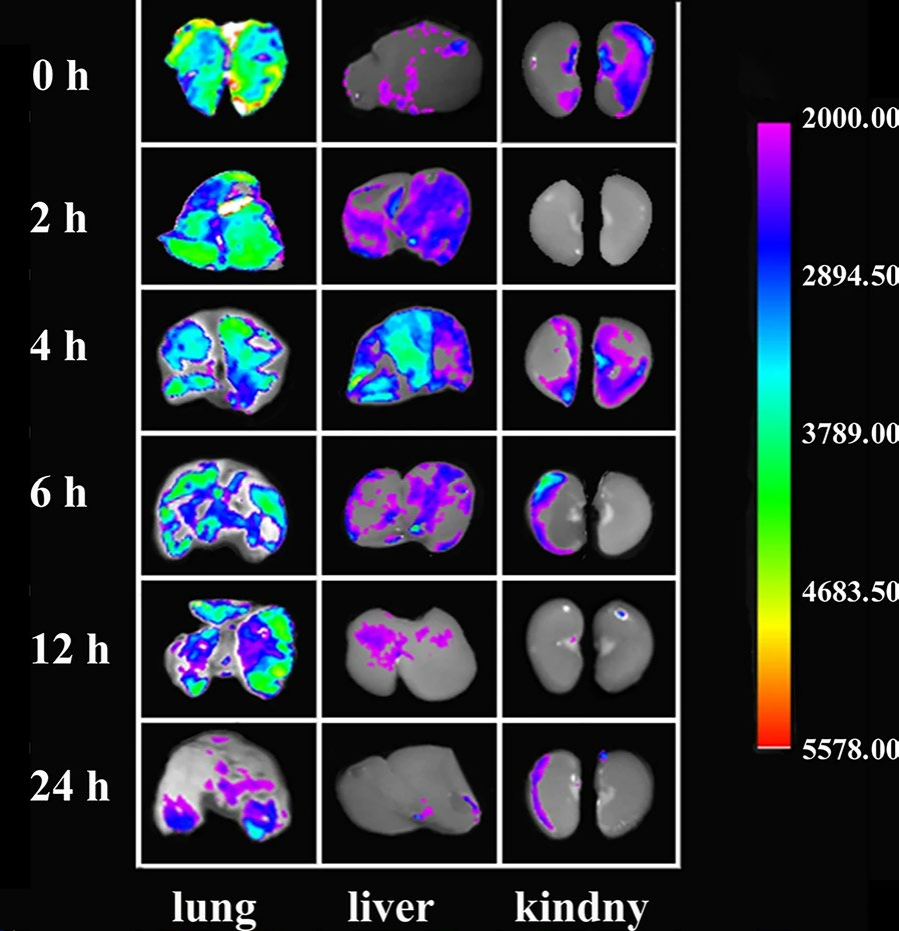
Tissue distribution of RBITC-QU-Nanogel in rats after aerosol inhalation. After aerosol inhalation of RBITC-QU-Nanogel (3 mg/mL) for 20 min, the lung, liver, and kidney were observed at predetermined time points (0, 2, 4, 6, 12 and 24 h) (Reproduced with permission under the terms of CC-BY license [49])

Inhaled drugs are delivered directly to the location of the disease [49], as portrayed in Fig. 2, which reduces the adverse effects caused by oral or intravenous drug delivery methods. The respiratory tract is directly connected to the external environment, and the lungs, as the main organs of the respiratory system, are responsible for ventilation and air exchange. Therefore, inhalation therapy is more effective for the treatment of respiratory system disorders compared to that for other internal organs. Systematic adverse effects are the main factors that limit the application of drugs, such as photosensitivity due to PFD [50] and diarrhea induced by Nintedanib [51]. Inhalation therapy allows localized treatment while minimizing unnecessary adverse effects, which is appealing.

Inhalation therapy provides rapid clinical responses, leading to immediate control of the disease. It has been widely used for a long time for treating COPD and asthma, with inhaled bronchodilators and glucocorticoids being the cornerstones of treatment. The efficacy of inhalation therapy is associated with the ability of the drugs to interact with cellular receptors in the airway, which are abundant and complicated. For example, autonomic receptors on airway smooth muscles are the target of β2 agonists and cholinergic receptor antagonists [52]. Based on the findings of many studies, an increasing number of novel receptors are being developed as targets for intervention in PF.

Inhalation therapy enhances drug utilization by mitigating hepatic first-pass metabolism and circumventing gastrointestinal malabsorption. Based on the theory of bioequivalence, pulmonary delivery of aerosols is noninvasive, and the effective dose is considerably lower than the oral or intravenous dose. Taking inhaled corticosteroids (ICS) as an example, ICS, such as budesonide and fluticasone propionate, have the following advantages: high rate of lung deposition, optimized lung residence time, rapid systemic clearance, and few adverse effects [53]. PFD is a small-molecule drug taken orally to treat IPF. Rasooli et al. found that the nebulized inhalation of PFD for 14 days was successful in treating PF induced by paraquat (PQ) in rats. The study suggested that nebulized inhalation of PFD is an effective treatment for IPF, and this method decreased the required drug dosage by nearly 10 times [54]. Similarly, the inhalation of Nintedanib demonstrates commensurate potential [55].

Inhalation therapy can be more convenient for patients, especially those who have difficulty swallowing pills or require frequent medication. Inhalers are portable and can be used anywhere, which helps patients adhere to their treatment regimen. Inhalation therapy can also be tailored to the needs of patients, with different devices and formulations available to facilitate optimal drug delivery.

Overall, inhalation therapy has several advantages over traditional drug delivery methods for respiratory diseases. It provides targeted and localized treatment, rapid clinical response, better drug utilization, and greater patient convenience. With the development of new inhalable drugs and delivery systems, inhalation therapy might become more effective and widely used in the future.

### Challenges

**Fig. 3:**
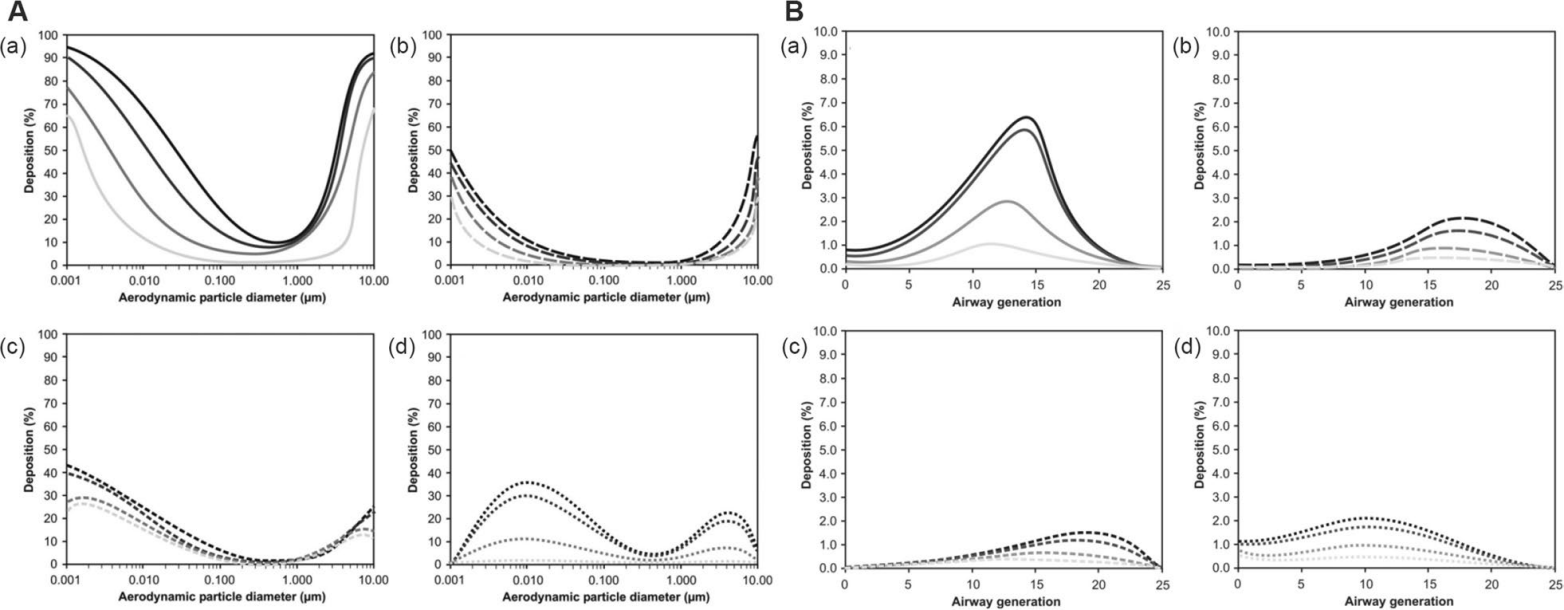
The relationship between aerodynamic diameter and lung deposition. **A** Investigating the relationship between particle size and deposition in the nose and lungs (**a** total deposition, **b**, **c** extrathoracic bronchial deposition, and **d** alveolar deposition). **B** Investigating the relationship between particle size and deposition in the mouse and lungs (**a** total Deposition, **b**, **c** extrathoracic bronchial deposition, and **d** alveolar deposition) (Reproduced with permission under the terms of the CC-BY license [56])

**Fig. 4:**
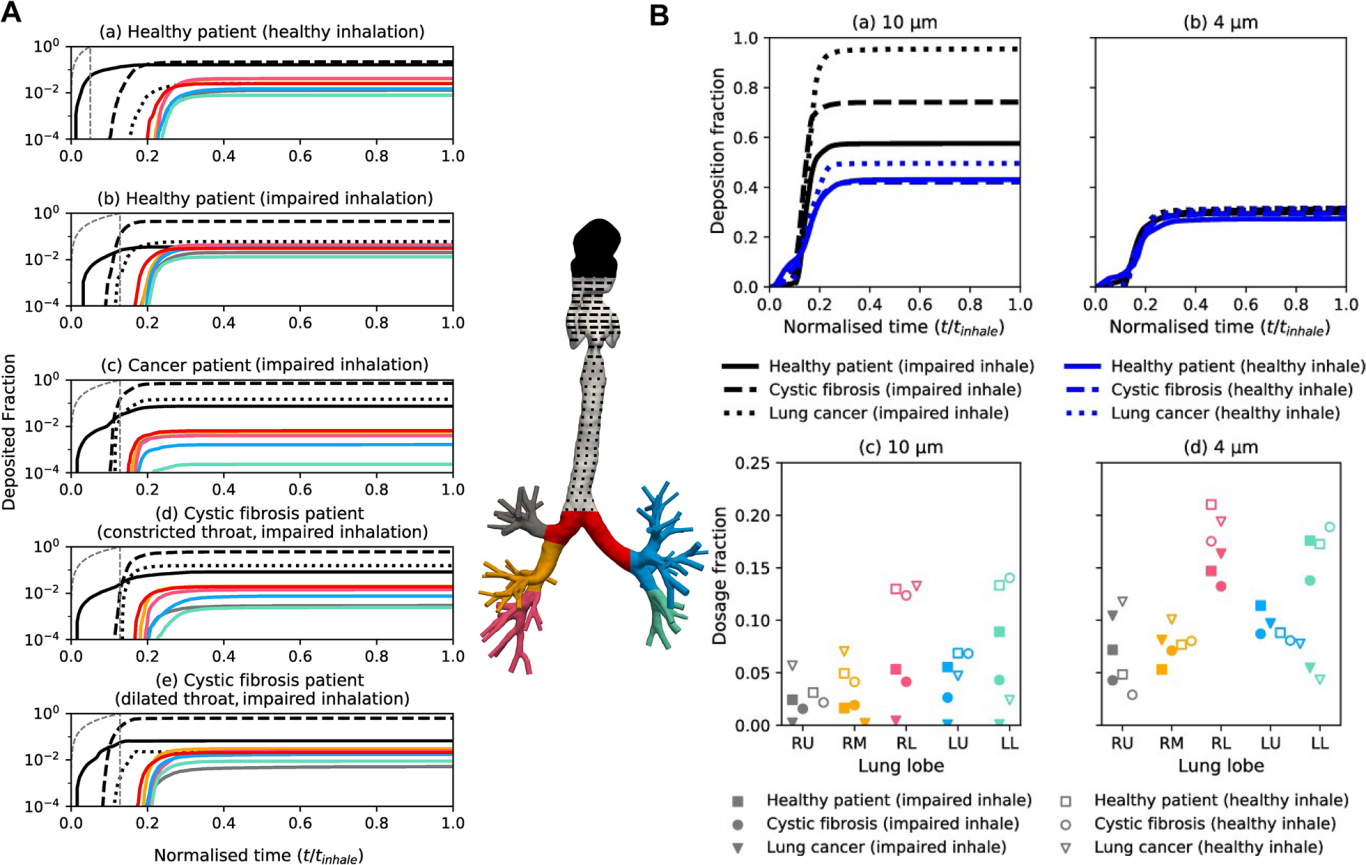
Chronic lung diseases impact lung deposition rates, posing challenges for inhalation therapy. **A** Deposition is shown on a log scale to allow closer analysis of lobar deposits. **B** Comparison of deposition under different breathing profiles for 10 µm, and 4 µm particles (Reproduced with permission under the terms of the CC-BY license [61])

**Fig. 5:**
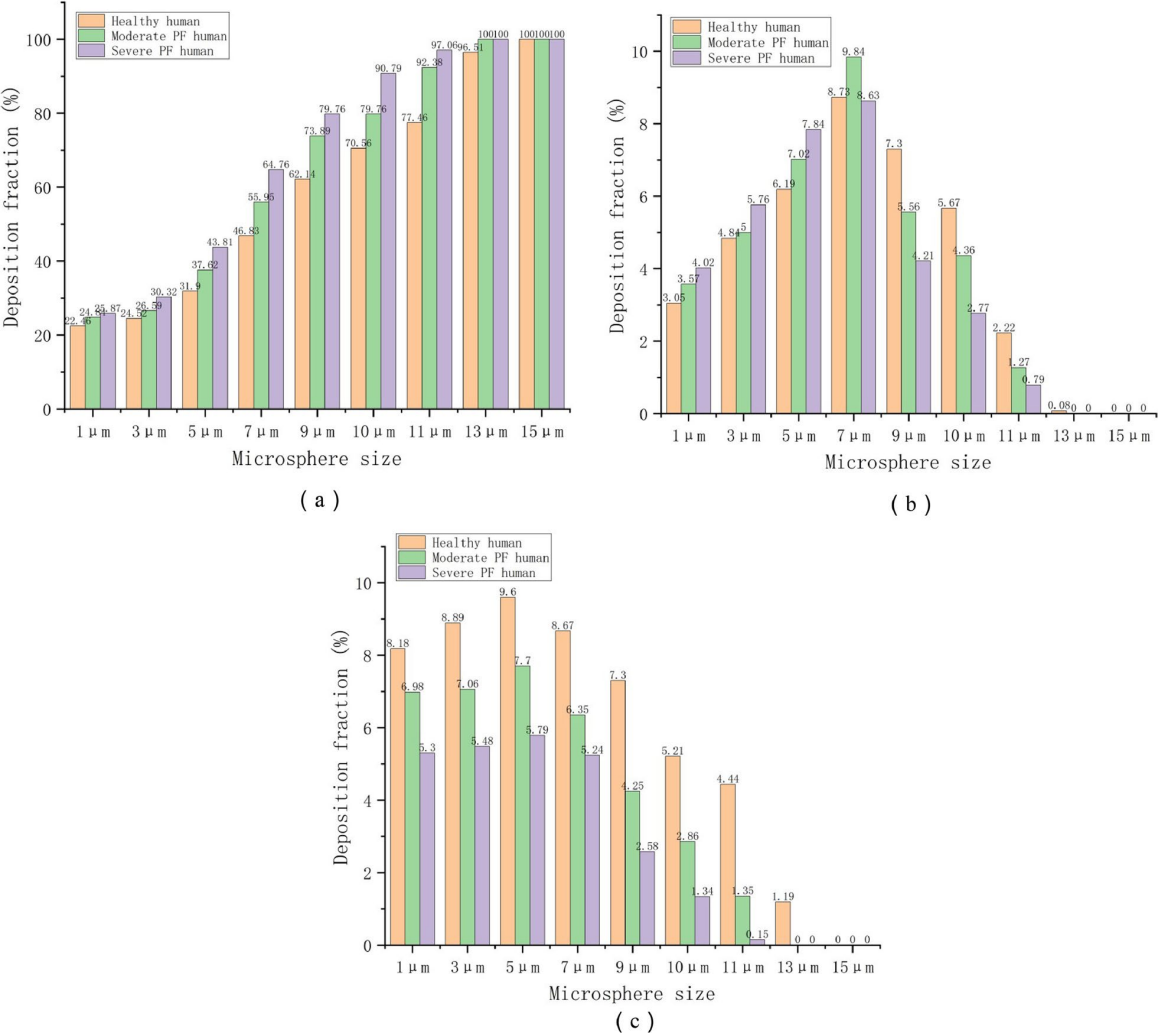
The impact of different severity levels of pulmonary fibrosis on lung deposition rates. **a** Upper respiratory tract, **b** main airway and G1 bronchus, **c** G2 and G3 bronchus (Reproduced with permission under the terms of the CC-BY license [62])

Enhancing the rate of drug deposition is a big challenge for inhalation therapy. The rate of deposition depends on the physicochemical properties of the aerosol. The deposition region of aerosols in the lungs is correlated with the particle size of the aerosols [56], as portrayed in Fig. 3. The human respiratory tract can be divided into three regions, including the nasopharynx, the conducting airway (tracheobronchial section), and the respiratory airway (alveolar ducts, alveoli, etc.). Inhalation therapy aims to achieve deposition in the respiratory airway region [57]. Aerosols with aerodynamic diameters less than 5 µm and 3 µm have the greatest potential for deposition in the lungs of adults [33] and children [58] respectively. Three physical molecular motion mechanisms dominate the different deposition patterns, including inertial impaction, gravitational sedimentation, and Brownian motion [59]. Aerosol particles larger than 1 μm experience inertial impaction when the flow rate is high, mainly at the oropharynx or the bifurcation of the central airway. This reduces the delivery of aerosols to the lower airway [60]. Gravitational sedimentation effects occur in small airways and alveoli when the flow rate is low, especially for particles smaller than 0.5 μm [60]. Brownian motion predominates when the aerodynamic diameter of the aerosol is less than 1 μm, but it is difficult to achieve Brownian motion with aerosol particles of drugs. When the particles are too small, the aerosols can be easily exhaled, thus, reducing the deposition rate in the lungs. Chronic lung diseases impact lung deposition rates, posing challenges for inhalation therapy [61], as portrayed in Fig. 4. The particles deposited in the lower airway gradually decrease with the aggravation of PF, which challenges the kinetic diameter of the inhaled drug particles. Qin et al. found that significantly fewer particles were deposited in the lungs of PF patients compared to that deposited in the normal airway under equivalent conditions, based on the calculations of the fluid dynamics of simulated particles in the airway of PF patients [62], as portrayed in Fig. 5.

**Fig. 6:**
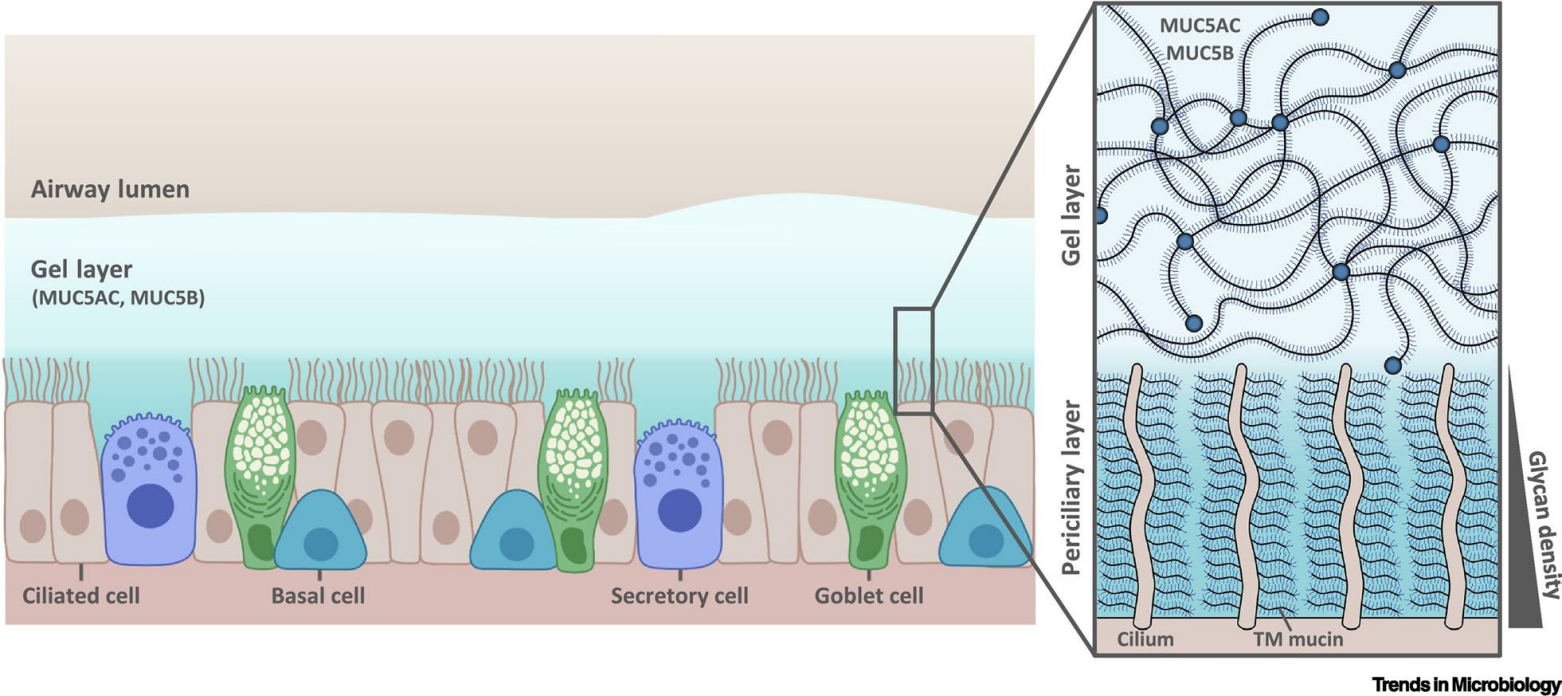
The mucus clearance mechanism in the bronchial and bronchiolar regions (Reproduced with permission under the terms of the CC-BY license [63])

Lung clearance mechanisms, including mucus cilia clearance and macrophage clearance, are major factors that affect the rate of aerosol deposition in the lungs. The mucus clearance mechanism primarily occurs in the bronchial and bronchiolar regions, which are mainly composed of mucins secreted from the airway surface [63], as portrayed in Fig. 6. The two primary mucins involved in this process include human MUC5B mucin (MUC5B) in the proximal airway and human MUC5AC mucin (MUC5AC) in the proximal and distal airways. These mucins combine to form mucus “blankets” or “flakes” that move upwards at a specific rate, which helps in removing particles that adhere to the mucus [64]. The clearance mechanism of alveolar macrophages (AMs) primarily operates in the peripheral airways of the lungs and is mainly responsible for the phagocytosis and clearance of particles ranging from 0.5 to 10 µm. After the particles are phagocytosed, AMs move toward the middle and upper airways through cellular movement to be excreted by mucus clearance mechanisms [65]. Therefore, designing a drug with a suitable aerodynamic diameter is necessary for achieving the optimal deposition rate in the lungs and the efficacy of inhalation therapy.

The biological barrier represents the second challenge for inhalation therapy. Lung diseases, such as IPF and CPF, disrupt the biological barrier and create technical obstacles for inhalation therapy, and the situation worsens because of the already existing physiological barrier of the lungs. The gas-blood barrier (GBA) is an important physiological process that not only maintains normal ventilation but also serves as the primary barrier, preventing the entrance of particles [66]. The GBA consists of several layers, including the fluid layer (pulmonary surfactant secreted by AT2 cells), the alveolar epithelial cell layer, the matrix layer (collagen fibers, fibroblasts, etc.), and the capillary layer (capillary basement membrane and endothelial cell layer), which exert different barrier effects on drug delivery. Pulmonary surfactant is a lipid-surfactant-specific protein complex that maintains alveolar mechanical stability and adhesion and promotes the expulsion of antigens, bacteria, viruses, and inhaled particles. It is an efficient drug carrier [67]. However, using it effectively is challenging. From a pathological perspective, the biological barrier disrupted by diseases makes inhalation therapy more challenging. In IPF, repeated epithelial cell injury, disordered proliferation of ECM and fibroblasts, and other events disrupt the matrix layers in AECs and GBA, leading to interstitial fibrosis and blocking the entry of drugs from the air into the bloodstream [68]. In patients with CPF, the CFTR gene variant impairs mucus clearance, leading to the accumulation of thick sputum that obstructs the airway and impedes drug transport [69].

**Fig. 7:**
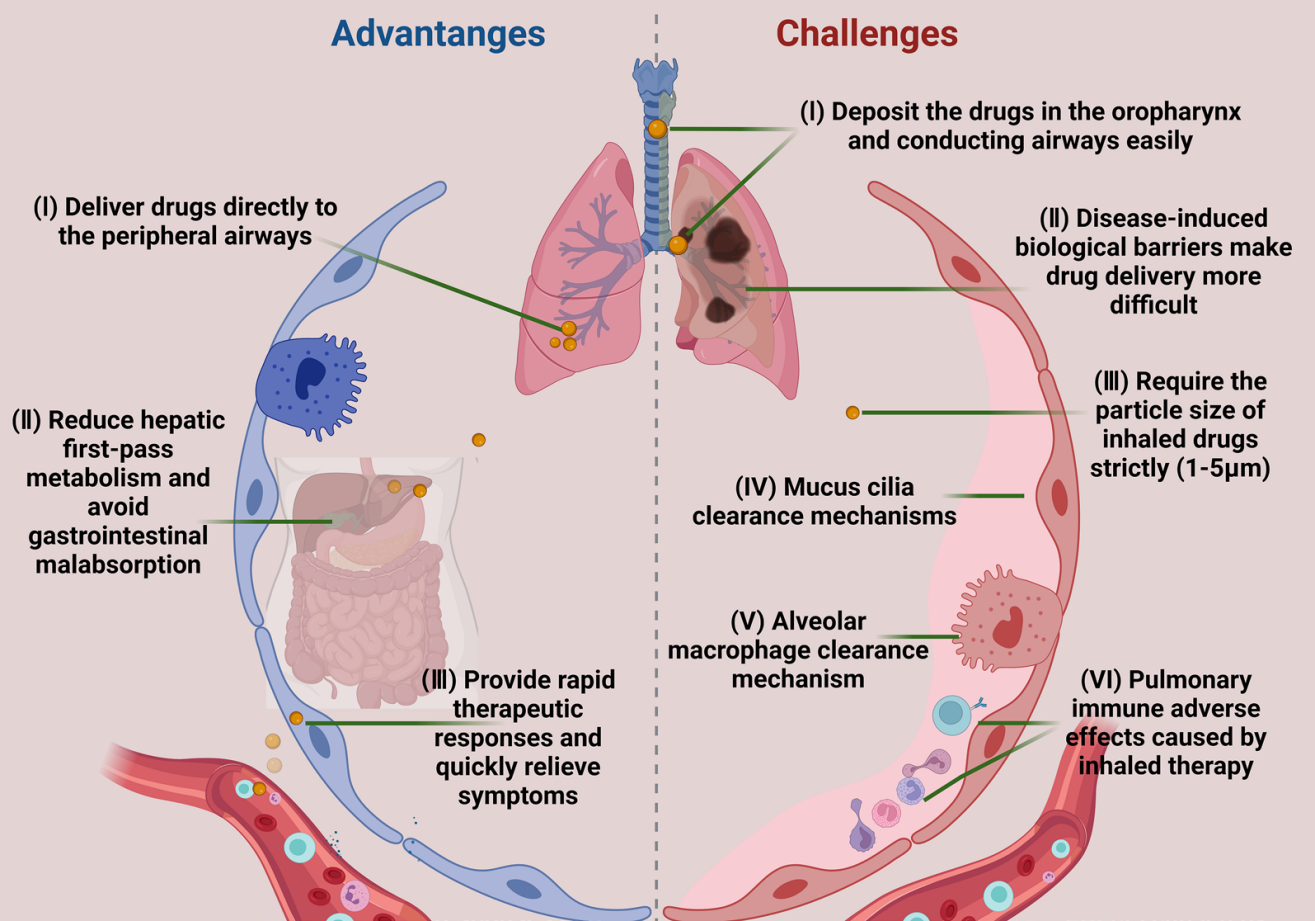
The advantages and challenges of inhalation therapy. This figure is created by BioRender and has confirmed publishing and licensing rights, with agreement number DO256A1GVN

Pulmonary immune adverse effects caused by inhalation therapy can be a significant challenge, as the safety and efficacy of the drugs are important. The lung possesses a powerful immune system composed of innate and adaptive immune cells, which can detect and phagocytose antigens and activate antigen-specific B cells and T cells for the specific clearance of antigens [70]. When antigens invade, innate immune cells, such as epithelial cells, macrophages, dendritic cells (DCs), and neutrophils, recognize and phagocytose them for clearance. However, the innate immune system alone might not be sufficient for antigen clearance. Adaptive immune cells subsequently exert an immune effect. Antigens activate antigen-specific B cells, and antibodies neutralize the antigen for clearance. Also, after recognizing pathogens, certain cells (mainly lung macrophages and DCs) act as antigen-presenting cells (APCs), and they present the major histocompatibility complex class I (MCH I) and activating antigen-specific T cells (CD4^+^T cells and CD8^+^T cells) for the specific clearance of the antigen, thus preventing the replication and spread of the pathogen [70, 71]. Since the lungs possess an extremely powerful immune system, the immune system might exert clearance effects to reduce the deposition rate in the lungs after the entry of inhaled drugs. This might trigger an excessive or adverse immune response, yielding counterproductive results [72]. Therefore, synthesizing drugs that can avoid adverse immune effects and exert immunomodulatory effects is important to ensure the efficacy of inhalation therapy. The advantages and challenges were portrayed in Fig. 7.

## Novel inhalation formulations in pulmonary fibrosis

Inhalation therapy can be highly effective in various lung diseases. However, addressing the challenges associated with inhalation therapy requires continuous research and the development of new strategies. One of the primary research objectives involves reversing the pathological processes involved in several lung diseases, particularly PF. Novel inhalation therapy is a promising strategy to achieve this goal. Therefore, reviewing the current research on inhalation therapy for treating PF and generating ideas for future studies are essential. Table 2 has depicted the recent research findings on various inhalation therapies for treating for PF.

**Table 2:** Examples of successful novel inhalation drug delivery pulmonary system interventions for pulmonary fibrotic diseases

Author, year	Indications	Cargo	Excipient	Surface modification	Particle size (nm)	In vivo/vitro	Administration	Key results
Seo et al. 2016 [116]	BLM-PF	Tac	Alb-NPs	NA	182.1 ± 28.5	C57BL/6	Intratracheal micro-spraying by microsprayer aerosolizer	The inhaled Tac Alb-NPs group displayed the better anti-fibrotic efficacy than intraperitoneal administration
Lee et al. 2016 [117]	BLM-PF	Tac	PLGA-NPs	CTS	320 ± 9.0	C57BL/6	Intratracheal micro-spraying by microsprayer aerosolizer	The inhaled Tac Alb-NPs group reduced the collagen deposition more than oral Tac administration
Elkomy et al. 202 [118]	BLM-PF	NFD	PLGA-NPs	CTS	188.20 ± 10.34	Wistar rats	Intratracheal micro-spraying	The inhaled NFD-CFS-PLGA-NPs group reduced the levels of oxidative stress and fibrosis-related indicators
Chen et al. 2022 [49]	PQ-ALI	QU	Nanogel	NA	QU-Nanogel: 61.87 Blank-Nanogel: 77.87	SD rats/A549-GFP	Nebulization by ultrasonic atomizer	The inhaled QU-Nanogel group acted the down-regulation effects of inflammation cytokines and oxidative stress
Bai et al. 2022 [122]	BLM-PF	siIL11	PLGA	PEG	NA	C57BL/6	Nebulization by vibrating mesh nebulizer	The inhaled NPs group significantly improved the pulmonary function test of mice
Zhang et al. 2022 [123]	BLM-PF	mMMP13	NPs	PEG	261.6 ± 2.558	C57BL/6	Nebulization by air compressor nebulizer	The inhaled NPs group significantly improved PF by accelerating alveolar reepithelialization
Garbuzenko et al. 2017 [134]	BLM-PF	PEG2, siMMP3, siCCL12, HIF1A	NLCs	NA	Empty NLC: 250 ± 30 With siRNA: 400 ± 50	SKH1-hr hairless mice	Nebulization by collison nebulizer	The group of NLCs containing PEG2 combined with three siRNAs achieved the best therapeutic effects
Hu et al. 2018 [108]	BLM-PF	Curcumin	LPMPs	NA	NA	SD rats	Dry powder inhalation by a handheld, dry-powder, breath-activated inhaler device	The curcumin LPMPs possessed the available size (3.12 μm) and inhibited PF by reducing the expressions of TNF-α, NF-κB p65 and MMP9
Hemmati et al. 2021 [109]	BLM-PF	Curcumin	NPs	NA	275	SD rats	Nebulization	The group of nano-curcumin of 200 μg/kg attained the best anti-inflammatory and protective effects against BLM-induced PF
Chen et al. 2022 [110]	Radiation pneumonitis	Curcumin	MPDA	NA	290.73 ± 29.73	SD rats/BEAS-2B	Intratracheal administration	The group of curcumin-MPDA exerted anti-fibrosis effect through good anti-oxidation and anti-inflammatory ability
Nafee et al. 2014 [139]	CF	QSI	SLNs	NA	NA	NA	Nebulization by ultrasonic atomizer	The group of QSI-SLNs (< 100 nm) could inhibit more
Moreno-Sastre et al. 2016 [140]	CF	Tb	NLCs	NA	250	BALB/c OlaHsd mice	Intratracheal micro-spraying by microsprayer aerosolizer	virulence factor pyocyanin
Garbuzenko et al. 2019 [138]	CF	luma/lva	NLCs	PEG	282.2 ± 8.5	Wistar rats	Nebulization by one-jet collison nebulizer	The group of luma/lva-NLCs (128.04 ± 1.58 nm) significantly reduced the affected regions in mice lungs
Dinh et al. 2020 [152]	BLM-PF	NA	LSC-Exo	NA	78.4 ± 2.5	SD rats	Nebulization by air compressor nebulizer	The group of LSC-Exo could attenuate PF by reestablishing normal alveolar structure and decreasing myofibroblast proliferation
Jiang et al. 2019 [145]	BLM-PF	H_2_	NA	NA	NA	Wistar rats	Exposed to either air or 2% H_2_	The H_2_ inhalation reduced ROS production and inhibited TGF-β Pathway reverses the process of EMT
Toshiyuki et al. 2021 [146]	BLM-PF	H_2_	NA	NA	NA	C57BL/6 mice	Exposed to either air or 3.2% H_2_	Inhalation of H_2_ significantly reduced M_2_-biased macrophages and pro-inflammatory cytokines

*ALI* acute lung injury, *Alb-NPs* albumin nanoparticles, *BLM* bleomycin, *BEAS-2B* bronchial epithelium cell line, *CTS* chitosan, *CF* cystic fibrosis, *CSP7* caveolin-1 scaffolding domain 7-mer peptide, *EMT* epithelial–mesenchymal transformation, *H*_*2*_ hydrogen gas, *LPMPs* large porous microparticles, *luma* lumacaftor, *lva* ivacaftor, *LSC-Exo* lung spheroid cell-exosomes, *MPDA* mesoporous polydopamine nanoparticles, *mMMP13* messenger RNA of MMP13, *NLCs* nanostructured lipid carriers, *NFD* nifedipine, *NPs* nanoparticles, *N*_*2*_ nitrogen gas, *PLGA* polylactic acid-hydroxyacetic acid, *PEG* polyethylene glycol, *PQ* paraquat, *QU* quercetin, *QSI* quorum sensing inhibitor, *RLI* radiotherapy lung injury, *SD* Sprague–Dawley, *siIL11* siRNA-IL11, *siMMP3* siRNA-MMP13, *siCCL12* siRNA-CCL12, *siHIF1A* siRNA-HIF1A, *SLNs* solid lipid nanoparticles, *VMN* vibrating mesh nebulizer, *Tac* tacrolimus, *Tb* tobramycin

### Based on basic experimental research

#### Agonists or inhibitors

The Nrf2 pathway and its downstream oxidative-antioxidant signaling pathways, as well as, the phosphatidylinositol 3-kinase/protein kinase B (PI3K/AKT) inflammatory signaling pathways, strongly influence the development and progression of PF [73, 74]. Enhancing the antioxidant capacity and blocking inflammatory pathways can help in alleviating PF. Therefore, activators of antioxidant factors and inhibitors of inflammatory proteins are commonly used in research. Nrf2 is a central regulator of cellular antioxidant response and redox balance recovery, and the application of Nrf2 activators can effectively inhibit PF and can significantly reduce the symptoms of PF in vivo and in vitro [75–77]. Dimethyl fumarate (DMF) is a first-generation Nrf2 activator. Muralidharan et al. prepared DMF NPs and MPs by spray drying (SD) and using co-SD techniques. They determined the geometric diameter, morphology, moisture content, and microscopic spectrum using scanning electron microscopy (SEM), X-ray powder diffraction, Karl Fischer titration (KFT), confocal Raman spectrometer (CRM), and next-generation impactor to evaluate the deposition of the particles in the airway [78]. They found that the particle size of DMF NPs and MPs was stable, and the airway deposition rate was also high. Liu et al. constructed a ROS-responsive nanoplatform (DSPE-PEG_2000_-ROS-sensitive linker thioketal [TK]) and loaded DMF onto the platform. The researchers made BLM-modeled PF mice (modeling from the 10th day, once every 3 days, for 14 days) inhale the above-mentioned drugs. They found that the inhalation of these drugs helped maintain the balance of superoxide dismutase (SOD) and ROS through the activation of the Nrf2/HO-1 signaling pathway significantly reduced the PF score of mice, and reduced the number of M2 macrophages and the secretion of TGF-β; which in turn improved the condition of the mice with PF [79]. Another study showed that the inhalation of a PI3K inhibitor (CL27c) significantly improved the lung function, such as the total FVC, lung capacity, and deep inhalation volume [80], of BLM-modeled PF mice. BLM administration via the airway is the most used animal model for PF [81].

However, this model still has certain limitations and cannot fully replicate the pathological process of human IPF. Firstly, in most animal species, a single dose of BLM induces a pronounced temporal progression characterized by initial pulmonary injury followed by fibrotic development. During this process, fibrosis typically undergoes rapid progression and reaches a peak within a few weeks, subsequently undergoing spontaneous resolution [81]. In contrast, human IPF is a chronic and progressive disease that may take months or even years to develop and progress to irreversible fibrosis [6]. Additionally, the response to therapeutic interventions may differ between mice and humans. In numerous animal studies, drug interventions frequently target the pre-fibrotic stage, demonstrating preventative capabilities against fibrotic progression. Nevertheless, human fibrosis exhibits a covert nature whereby patients often enter the fibrotic phase prior to receiving a definitive diagnosis. Moreover, assessing the true anti-fibrotic potential without suppressing inflammation is one of the current limitations in this regard. BLM can upregulate the expression of specific transport proteins to induce drug efflux in experimental mice, reducing the exposure of experimental drugs and potentially decreasing the reliability of preclinical outcomes [82]. Therefore, it is necessary to develop animal models that better replicate the pathological processes of human IPF to accurately evaluate and develop drugs for this disease. Such animal models should be able to simulate the chronic progression, irreversible fibrosis, and associated inflammatory and cellular proliferation processes of IPF.

Along with the agonists or inhibitors of proteins on related signaling pathways, inhibitors of specific proteins are also important targets for the treatment of IPF. Some studies have found that αvβ6 integrin is significantly upregulated in IPF patients and promotes the progression of IPF by activating TGF-β [31, 83]. Therefore, αvβ6 inhibitors are important targets for intervention in IPF [84, 85]. GSK3008348 is a small-molecule αvβ6 inhibitor that can strongly inhibit the secretion of TGF-β and decrease the rate of development of PF. In vivo studies have shown that the inhalation of GSK3008348 can have this effect [86, 87]. These studies showed that (i) inflammatory and oxidative stress pathways are involved in the pathogenesis of IPF and (ii) the inhalation of specific signal pathway agonists/inhibitors might help PF intervention. These results suggested that multiple signaling pathways influence the occurrence and development of PF. Therefore, the activation or inhibition of specific signaling pathways is an important means of treating PF.

#### Nanomedicine platform

Nanomedicine refers to the use of nanoscale particles produced by nanofabrication techniques as drug delivery vehicles or active ingredients in pharmaceutical formulations [88]. It includes the direct application of nano-sized drugs and nanocarrier systems. The former involves the use of nanoscale precipitation or ultrafine grinding to prepare drug NPs, while the latter involves combining drugs with carrier materials through dissolution, dispersion, encapsulation, adsorption, coupling, etc., to form nanodispersions. These nanodispersions mainly consist of polymers [89], lipid-based NPs [90], dendritic macromolecules [91], microspheres [92], and magnetic NPs [93], which are designed with different building elements and internal structures. The external size, internal structure, or surface structure of the final product or carrier material of nanomedicine are in the nanoscale level (i.e., < 100 nm), or the size of the particles is below 1000 nm with a clear size effect [94]. Along with drugs of nanometer-scale size, particle-shaped drugs of micrometer-scale size are also included in this section.

**Fig. 8:**
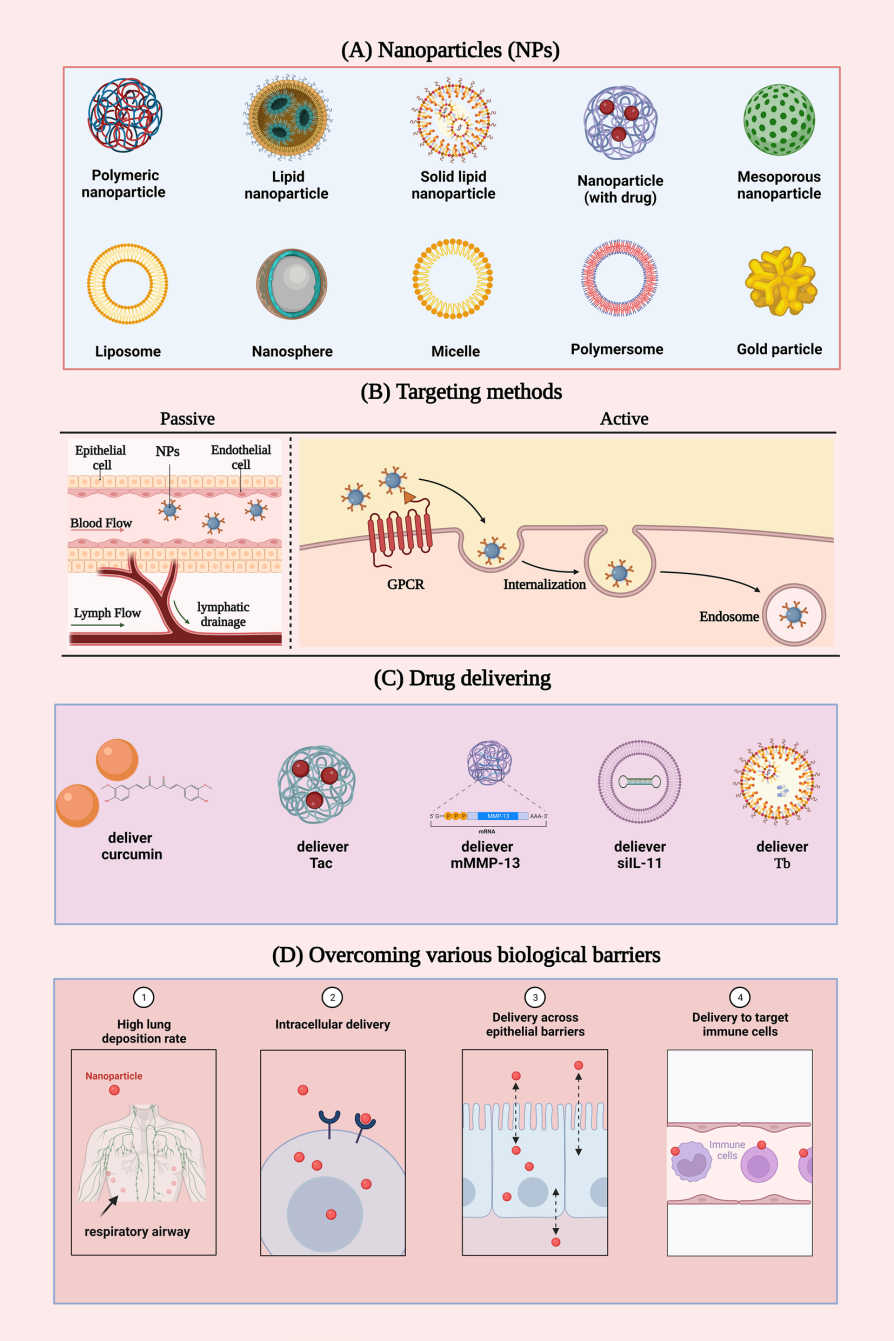
Advantages of inhalation therapy of nanomedicine for the treatment of pulmonary fibrosis. This figure was created by BioRender and has confirmed publishing and licensing rights, with agreement number QH256A1W2H

**Fig. 9:**
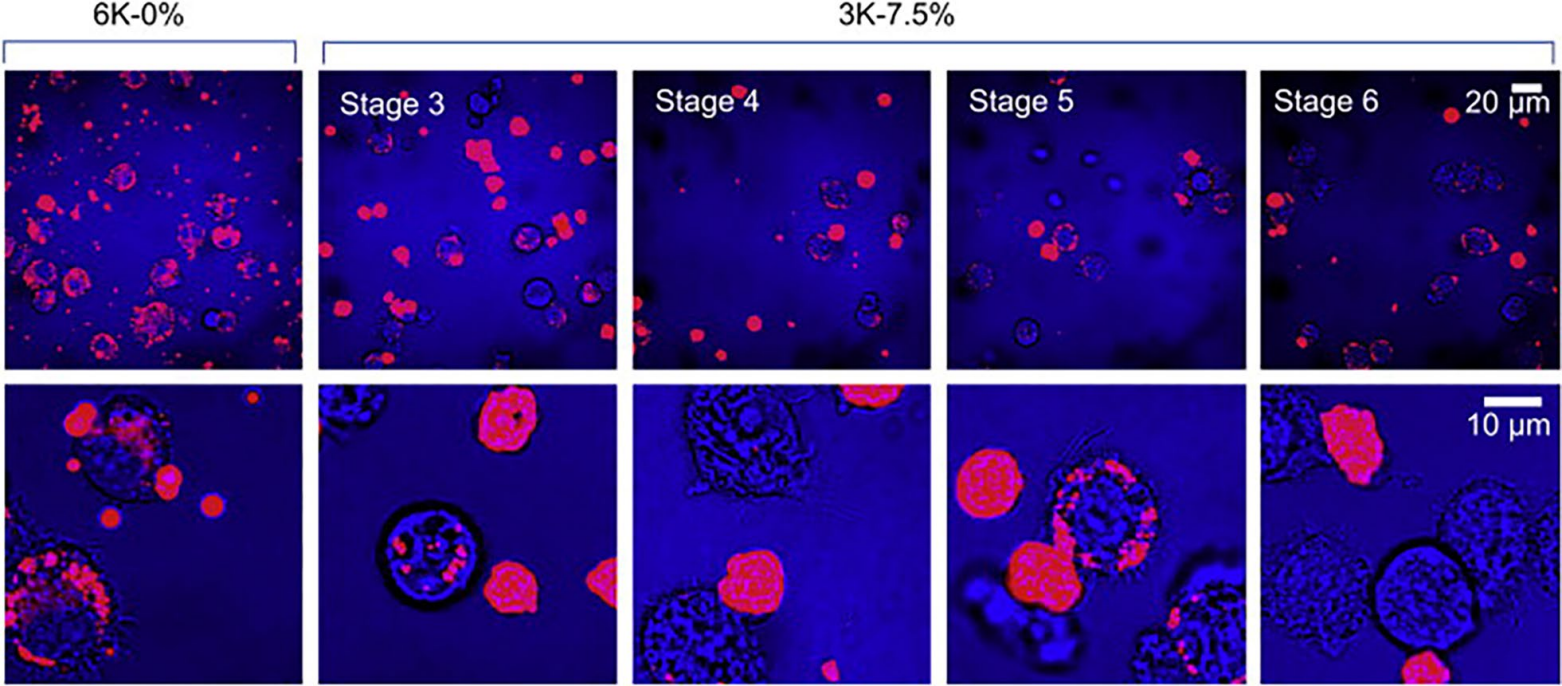
Macrophage uptake of non-porous (6K-0%) and highly porous (3K-7.5%) PLGA microparticles. Red fluorescence images of microparticles were superimposed with transmission images (blue) of macrophages and microparticles (Reproduced with permission under the terms of the CC-BY license [97])

**Fig. 10:**
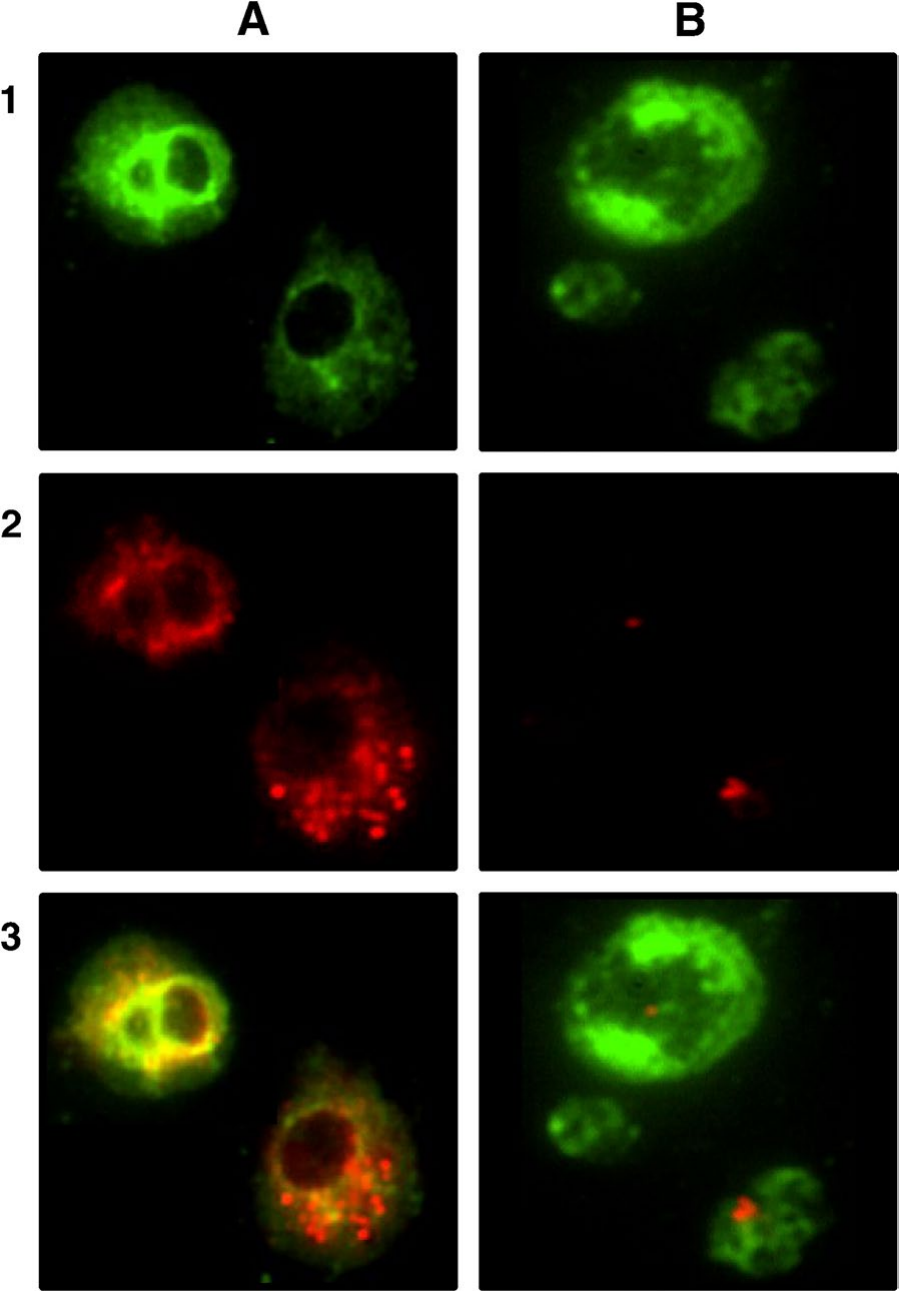
NPs with surfaces modified by polyethylene glycol (PEG) avoid being taken up by AMs. **A** Uptake of microparticles by rat alveolar macrophages 1 h after incubation with PLGA. **B** Uptake of microparticles by rat alveolar macrophages 1 h after incubation with PEG-PLGA (Reproduced with permission under the terms of the CC-BY license [99])

Several studies have investigated the application of inhaled nanomedicine in recent years for pulmonary delivery via inhalation devices such as nebulizers, pMDI, and DPI. Compared to conventional inhaled pharmaceutical therapy, nanomedicine has several unique advantages, as portrayed in Fig. 8. First, MPs (1–500 nm) and NPs can achieve higher lung deposition rates because they are considerably smaller. Second, NPs are more likely to cross the biological barrier. AMs are the key cells for particle clearance [95], and their ability to phagocytize NPs is relatively lower, which facilitates longer lung retention time and drug release. An in vivo study showed that rat AMs engulfed fewer particles < 1 μm than they engulfed larger particles (1–5 μm) [96, 97], as depicted in Fig. 9. Third, the properties of the NPs and surface modifications can further reduce particle uptake by AMs. For example, particles containing cholesterol and sphingolipids, NPs with relatively neutral surface charges, soft and porous particles, soluble and hydrophilic particles, and NPs with surfaces modified by polyethylene glycol (PEG) or dipalmitoyl phospholipids (DPPC) avoid being taken up by AMs [98, 99], as illustrated in Fig. 10. Therefore, MPs and NPs can be effective in inhalation therapeutic interventions for PF as they possess the above properties.

##### Drug nano-size systems

The direct conversion of traditional Chinese medicine (TCM) monomers into MPs and NPs has been widely performed in basic research on PF intervention. Based on various active ingredients in TCM, such as curcumin, cryptotanshinone, and tetrandrine, have shown anti-PF effects by targeting multiple pathways [100–105]. Also, several studies developed dry powder and nebulized solutions of TCM monomers for PF intervention, such as the dry powder preparation of salvia polyphenolic acid and nebulized solution of Panax ginseng total saponin [106, 107]. Curcumin (*Curcuma longa*) is an effective component in TCM. Hu et al. [108] developed a targeted drug delivery system for the treatment of PF using curcumin-loaded large porous MPs (curcumin-LPMPs) prepared through the W/O/W emulsion method. The curcumin-LPMPs were converted into dry powder capsules (30 mg/capsule) and inhaled using a dry powder inhaler. The researchers found that curcumin, used as dry powder alone and curcumin-LPMPs, exhibited antifibrotic effects, probably by inhibiting the NF-κB inflammatory pathway. Curcumin-LPMPs showed a better therapeutic effect due to their high lung deposition rate and lower macrophage uptake. Their findings suggested that the curcumin-LPMPs system might be used for the targeted therapy of PF [108]. By mixing β-cyclodextrin (40 mg) dissolved in deionized water and curcumin (12 mg) dissolved in acetone, Hemmati et al. prepared dry NPs (275 nm) through freeze-drying [109]. Inhalation of curcumin-NPs (50 μg/kg, 100 μg/kg, and 200 μg/kg) for 21 consecutive days significantly improved BLM-induced PF, as determined by a decrease in pulmonary inflammatory cell infiltration, lower levels of pulmonary hydroxyproline and cytokines (Tumor Necrosis Factor-alpha [TNF-α], Interleukin-10 [IL-10], and platelet-derived growth factor [PDGF]). Chen et al. loaded curcumin onto mesoporous polydopamine NPs (MPDA) through emulsion-induced interface polymerization. Polydopamine (PDA) is a type of polymer of melanin that has good biocompatibility and can be prepared easily. MDPA modified from PDA has abundant pore size structure, high drug-loading capacity, and can be more easily deposited in the lungs after inhalation. The resulting curcumin-MPDA (CMPN) was obtained by drying under a vacuum and exhibited antioxidative and anti-inflammatory effects; thus, they protected against radiation pneumonitis [110]. Radiation therapy is an effective method of cancer treatment. However, radiotherapy lung injury (RLI), including pneumonitis and PF, are common adverse effects that make many patients unsuitable for undergoing radiotherapy.

Although drugs of nano or micro size can improve efficacy and reduce adverse reactions, simply converting drugs into an inhalable form cannot guarantee the desired particle size and deposition rate. Specific excipients, ratios, and conversion methods need to be optimized to achieve an efficacious final inhalable drug. This was shown in the application of PFD and swellable MPs (SMPs). Kang et al. converted PFD into inhalable dry particles through co-spray drying, using methods such as spray-dried (SD) PFD, co-spray-dried PFD with L-leucine (SD-PL), co-spray-dried PFD with NaCl (SF-PN), and co-spray-dried PFD with mannitol PFD (SD-PM) to produce MPs [111]. The results showed that SD-PL (1:1) produced particles with the smallest size (Dv50: 4.28 µm) and the highest aerosol performance (3.85 ± 0.21 µm). Adding 10% l-leucine (LL) as excipient or SMPs can optimize aerosol performance, including flowability and expandability [112]. In a recent study, a carrier solution was prepared, which contained chitosan (CS) and LL in a mass ratio of 9:1. High-pressure homogenization and spray drying were performed to produce CS-SMPs [111]. The researchers showed that adding LL significantly enhanced the anti-humidity, flowability, yield, and nebulization efficiency of CS-SMPs. The particle size of CS-SMs was 464.4 ± 10.1 nm, and inhalation of the CS-SMPs prolonged the drug enrichment time in the lungs by up to 24 h in PF-rats. A similar anti-fibrotic effect was achieved with only 1/60 of the oral drug dose. This anti-fibrotic therapeutic effect might be attributed to the modulation of the TGF-β1/Smad3, STAT3, and sirtuin 3 (SIRT3) signaling pathways.

##### Drug nano-carrier delivery systems

Polymeric nanoparticles: Polymeric NPs (PNPs) are nanoscale particles composed of synthetic or natural polymers that are commonly used in inhalation therapy for PF. PNPs can be synthesized from various polymers, including polylactic acid (PLA), polylactic acid-hydroxyacetic acid (PLGA) copolymer, PEG, polycaprolactone (PCL), albumin, gelatin, alginate, CS, agarose, and others [113]. PNPs are favored and widely used for the inhalation therapy of PF. They enhance lung deposition, drug absorption, and other properties by changing the hydrophobicity and surface charge of the NPs depending on the type of polymer present.

Based on nebulizer inhalation: Nebulized PNPs are widely used for inhalation delivery in basic research [114]. Tacrolimus (Tac) is a potent immunosuppressant that might be effective in the treatment of PF. In 2006, Nagano et al. induced PF formation in mice by airway drip BLM and then performed continuous intraperitoneal injection of Tac for 7–14 days. They found that Tac improved PF through anti-inflammation and by enhancing pulmonary vascular permeability [115]. Following these findings, two studies investigated whether inhalation therapy with Tac-encapsulated NPs could alleviate PF. Seo et al. used Tac combined with albumin NPs (Tac Alb-NPs) for inhalation therapy [116]. They treated BLM-molded C57BL/6 mice with Tac Alb-NPs (182.1 ± 28.5 nm) via microspray aerosolizer for 18 consecutive days. The drug encapsulation rate was > 80%, and the lung deposition time was approximately 48 h. The pulmonary hydroxyproline (HYP) levels decreased significantly, inflammatory infiltration of lung tissue decreased, and PF was reduced in the treatment group. Additionally, the effect of inhalation administration was better than that of intraperitoneal administration. Lee et al. found similar results using chitosan (CTS) Tac PLGA-NPs [117]. They found that lung deposition after inhalation was prolonged to 96 h. Inhalation treatment reduced collagen deposition more than oral Tac administration. The prolonged lung deposition time of PLGA-NPs might allow a sustained release of Tac, leading to better therapeutic outcomes.

The cationic nature of CTS makes it an excellent bioadhesive agent and vehicle for delivering drugs, and modification of CTS enhances the viability of NPs and improves their biocompatibility and drug delivery performance. Elkomy et al. found that the optimal formulation of CTS-PLGA-NPs loaded with nifedipine (NFD) was 0.52% ω/υ PLGA, 1.5% ω/υ PVA, and 0.25% ω/υ CS [118]. The NFD-CTS-PLGA-NPs were delivered via continuous inhalation for 3 weeks using a microsprayer and were efficiently deposited in the lungs with a mass median aerodynamic diameter of 1.12 ± 0.28 µm and a fine particle fraction of 80.48 ± 8.46%. Treatment with NFD-CTS-PLGA-NPs was associated with lower levels of oxidative stress and fibrosis-related indicators, probably because of the inhibition of the TGF-β/β-Catenin signaling pathway.

Polymer-based nanogels, such as those made from CTS and alginate, are also widely used as drug delivery carriers and have advantages such as stable encapsulation, high bioavailability, and good water solubility [119]. In a recent study by Chen et al., the potent antioxidant and anti-inflammatory agent quercetin (QU) was co-constructed with alginate nanogel using Ca^2+^ and hydrogen bonding. The resulting QU-alginate nanogel was less than 100 nm and was used for the treatment of paraquat-induced acute lung injury (ALI) in rats via ultrasonic aerosol inhalation for 3–7 days [49]. The nebulized QU-alginate nanogel exhibited excellent antioxidant properties by modulating the levels of HO-1 and SOD and showed good anti-inflammatory properties by reducing the levels of TNF-α, IL-6, and IL-1β. Thus, treatment with nebulized QU-alginate nanogel effectively reversed ALI and inhibited the development of PF in rats.

Gene therapy is a promising approach for the treatment of various lung diseases, including IPF. Aerosol inhalation gene therapy includes various approaches, such as CRISPR/Cas9, messenger RNA (mRNA), small intervention (siRNA), plasmid DNA (pDNA), and protein-based gene therapy [120]. In IPF, the overproduction of IL-11 promotes fibroblast differentiation into myofibroblasts through the activation of the extracellular signal-regulated kinase (ERK) and SMAD pathways [121]. Therefore, anti-IL-11 therapy is a potentially effective treatment strategy for alleviating IPF. In a study [122], PEG-PLGA-NPs carriers loaded with siRNA were developed against IL11 (siIL11). The siIL11-PEG-PLGA-NPs exhibited a higher fluorescence signal in the lungs than PBS treatment, and a uniform distribution of NPs in each lobe was recorded. The NPs-treated group showed significant anti-fibrotic effects due to the inhibition of myofibroblast biomarkers, such as collagen type I alpha 1 chain (COLIA1) and actin alpha 2, smooth muscle (ACTA2). Additionally, the pulmonary function test (PFT) of the NP-treated mice, such as respiratory resistance and elastance, were significantly improved by blocking the ERK/SMAD pathway via intermittent nebulization of PF mice by using a vibrating mesh nebulizer six times (every 3 days). Gene therapy based on mRNA has also shown promising results in the treatment of IPF. In one study, the mRNA of matrix metalloproteinase 13 (mMMP13)-peptide-keratinocyte growth factor (KGF) was delivered to BLM-induced PF mice using PEG-NPs (mMMP13-KGF-PEG-NPs) via interrupted nebulization. The treatment was administered five times every 3 days using an air-compressed nebulizer. Compared to the nebulized group alone or the oral PFD group, the combined treatment group showed a significant improvement in PF by accelerating alveolar re-epithelialization, as determined by an increase in the ratio of aquaporin 5 (AQP5) to SP-C [123]. With further research and development, inhalation therapy based on genes might become a valuable technique in personalized medicine for the efficacious treatment of PF.

Based on dry powder inhalation: NPs are commonly used for CPF intervention through the inhalation of dry powder using various drying techniques. Inhalation of antibiotics is the preferred treatment strategy for managing CPF because it allows for maximum airway deposition and reduces the adverse effects of systemic drug exposure. However, the mucus barrier strongly hinders the effectiveness of inhaled antibiotics. Thus, many researchers have used nanocarriers to deliver multiple antibiotics across the mucus layer to achieve effective anti-infective action. Ungaro et al. loaded tobramycin (Tb) into CTS-NPs and poly (vinyl alcohol)-alginate/PLGA-NPs and converted them into dry powders by SD. The Tb-poly(vinyl alcohol)-alginate/PLGA-NPs were deposited at higher levels in the lower airways of the lungs in vivo, which indicated that PLGA NPs-based dry powder formulations are better for inhaled antibiotics [124]. Juntke et al. modeled human cystic fibrosis cells infected with *P. aeruginosa* using biofilm and found that planktonic bacteria were eradicated and the biofilm of the pathogen was significantly reduced after 1 h of intervention with ciprofloxacin-PLGA-NPs [125].

Lipid-based nanocarriers: The lipid NPs (LNPs) system, as described by Fonseca-Santos et al., consists mainly of solid lipid nanoparticles (SLNs) and nanostructured lipid carriers (NLCs). SLNs are the first generation of lipid nanocarriers (LNCs) and are generally prepared using physiologically compatible lipids or lipid-like substances as pharmaceutical excipients. SLNs possess the benefits of several nanocarriers, including PNPs, fat emulsions, and liposomes, making them highly suitable for delivering lipophilic drugs. They also have advantages such as high biocompatibility and good slow-release performance. However, they have certain disadvantages, including their low drug penetration ability, poor physical stability, and a low drug loading capacity of only 1–5%. Overall, while SLNs have satisfactory therapeutic potential, they might not be suitable for all drug delivery applications due to these limitations [126].

NLCs are second-generation lipid nanocarriers that can overcome the limitations of SLNs by improving the drug loading capacity and physical stability. NLCs are broadly classified into three types including the imperfect type, amorphous type, and multiple type. They have different degrees of lipid organization within the nanoparticles, which influences their physicochemical properties and drug-loading capabilities [127]. Lipid-drug conjugates (LDCs) and polymer-lipid hybrid NPs (PLNs) are important components of the NLC system. LDCs are lipophilic modifications of water-soluble drugs or other difficult-to-deliver compounds attached to lipids through covalent conjugation, which confers drug-forming properties to the drugs. The lipid materials used for conjugation are usually fatty acids, glycerides, phospholipids, etc. LDCs resolved the issue of the extremely low encapsulation rate of water-soluble drugs by SLNs and NLCs [128]. PLNs are optimized from SLNs by introducing negatively charged polymers to form drug-polymer complexes, which are then encapsulated in hydrophobic lipid materials. Polymer-lipid hybridization is usually achieved through hydrophobic interactions, van der Waals forces, electrostatic interactions, and covalent bonding. They have the advantages of liposomes and NPs.

It was encouraging to note that a substantial number of studies based on lipid-based nanocarriers for the inhalation treatment of IPF have achieved promising results. Prostaglandin E2 (PEG2) is a powerful pro-inflammatory factor with restricted immune-inflammatory and tissue repair effects in the lungs [129]. Its effectiveness as a potential drug was assessed for the treatment of IPF [130–133]. However, the limited half-life in the blood and lung distribution restricted the clinical application of PEG2 via systemic delivery. In 2017, Garbuzenko et al. loaded PEG2 and three siRNAs (MMP3, Chemokine (C-C motif) ligand 12 [CCL12], and Hypoxia-Inducible Factor 1 Subunit Alpha [HIF1A]) individually and along with NLCs (PEG2-siRNAs-NLCs), which were nebulized by a collision nebulizer for treating BLM-induced PF mice. The nebulization procedure followed a temporal pattern of intervention (twice a week for 3 weeks). The results showed that the most significant anti-fibrotic outcome was obtained with the combination of PEG2-siRNAs-NLCs [134], which was a successful application of the combined use of the delivery of NPs and gene therapy. INS1009 is a vasodilator, which releases hexadecyl-treprostinil (C16TR) from LNPs. Under the excitation of endogenous lung esterase, C16TR is converted to Treprostinil (TRE). TRE is a potent agonist of PEG2 and functions as an anti-fibrotic inhibitor of lung fibroblast proliferation. Corboz et al. found that in BLM-induced IPF mice, collagen fiber deposition decreased significantly after the mice inhaled INS1009, which imparted its effects in a dose-dependent manner [135].

The LNPs can improve bacterial multi-drug resistance, especially in patients with CPF. The multi-drug resistance mechanism of *P*. *aeruginosa* increases the dilemma of the antibiotic application of CPF [136, 137]. Several gene-based treatment methods have been explored by Garbuzenko et al., who developed PEGylated NLCs loaded with Lumacaftor (luma) and Ivacaftor (lva) to treat CPF. Inhaling these NLCs using a one-jet collision nebulizer resulted in a significant reduction in or the disappearance of lung lesions in mice with CPF [138]. However, the efficient delivery of drugs through the thick sputum to the lesion is a great challenge in clinical practice. *P*. *aeruginosa* produces virulence factors through quorum sensing (QS), and QS-inhibitors (QSIs) are effective against its virulence effects. Nafee et al. demonstrated that SLNs loaded with QSI (QSI-SLNs) could effectively penetrate the mucus, had high stability and anti-toxicity, and thus were safe [139]. These NPs (< 100 nm) were nebulized by an ultrasonic nebulizer, and their efficacy was higher than that of blank SLNs. NLCs also showed good performance in treating CPF, with Tb-loaded NLCs (Tb-NLCs) exhibiting a drug encapsulation rate of up to 93% [140]. The NPs were nebulized by MicroSprayer™ aerosolizer and showed uniform and long-lasting (until 48 h) in vivo distribution in the lungs of mice. Although the results of using NPs to deliver drugs for treating PF are promising, the stability of NPs after nebulization, particularly following drug loading, needs to be improved. To address this issue, Zhang et al. examined the stability of different formulations of LNPs loaded with mRNA (mRNA-LNPs) before and after nebulization using a VMN [141]. The researchers used a design of experiments strategy to optimize their study design. Their findings indicated that mRNA-LNPs delivered to the lungs by nebulization were relatively stable concerning changes in particle size, zeta potential, drug encapsulation rate, and intracellular protein. Their study provided valuable insights into the stability of NPs after nebulization which might facilitate the development of effective strategies for treating PF.

#### Other novel inhalation drugs

Besides the above-mentioned drugs, some new inhalation interventions may play an important role in treating PF. The inhalation of specific gases, such as hydrogen (H_2_), nitric oxide (NO), etc., can improve pulmonary lesions. H_2_ has antioxidant and anti-inflammatory effects, which can significantly alleviate diseases, such as ischemic brain injury, myocardial ischemia–reperfusion injury, and sepsis-induced liver injury [142–144]. A study found that the inhalation of H_2_ can reduce the secretion of pro-fibrotic factors TGF-β1 and TNF-α, weaken the process of epithelial-to-mesenchymal transition (EMT), and improve PF [145]. Another study reported that the continuous inhalation of hydrogen for 7 or 21 days can significantly improve macrophage polarization and pulmonary functions in BLM-induced PF [146]. Diethylenetriamine nitric oxide adduct (DETA/NO) is a donor with slow-release nitric oxide action. Chen et al. conducted continuous DETA/NO inhalation for 13 days in mice with BLM-induced PF and found that the inhalation of DETA/NO significantly reduced the number of myofibroblasts and type I collagen deposition [147].

Extracellular vehicles (EVs) are small structures that can be accepted by the extracellular environment. They can transport proteins, nucleic acids, and other substances and might be used to transmit information and regulate intercellular communication; thus, they can influence the behavior and functions of cells [148, 149]. The effectiveness of exosomes from various cell sources, such as exosomes from mesenchymal stem cells and macrophages, was assessed for treating PF [150, 151]. The intervention methods in those studies mostly included intravenous injections and airway instillation. Dinh et al. pioneered exosomes inhalation therapy for PF. In their study, mice with ALI and PF modeled by BLM/silica continuously inhaled exosomes from alveolar epithelial cells for 7 days through an air-compressed nebulizer. The results showed that the inhalation of exosomes significantly improved the lung function (compliance, residual volume, and the ratio of the forced expiratory volume in one second [FEV_2_] to FVC ratio [FEV_2_/FVC]) and reduced inflammatory factors, thus alleviating PF in these mice [152].

### Based on clinical trial of phase I/II

Many studies have investigated inhalation therapy for PF and determined the efficacy, mechanism of action, drug delivery methods, dosage, and pharmacokinetics of inhaled treatments in animal models of PF. Their findings provided important reference values and guidance for clinical trials. We searched for phase I and phase II clinical trials that reported results till March 15th, 2023, on ClinicalTrials.gov and found that some clinical studies using inhalation therapy achieved success in PF intervention.

Chronic cough, hypoxia, and decreased physical activity are common symptoms of PF. Inhalation therapy can alleviate these symptoms. Some preclinical studies found that supplementing with NO was beneficial in mouse models of fibrosis [147]. Christopher et al. significantly advanced inhalation therapy using NO for treating IPF patients. They conducted a phase-II randomized controlled trial (RCT), where they recruited 44 patients with fibrosing interstitial lung disease. They randomly divided the patients into two groups; one group received NO (45 µg/kg/h) via inhalation, while the other was a placebo group. They administered the treatment for 3 months and found that NO significantly improved the symptoms and physical activity levels of the patients [153]. The effects of the inhalation of carbon monoxide (CO) have also been examined in an RCT conducted with IPF patients. However, the results showed that 12 weeks of CO inhalation (100–200 ppm, twice a week) did not significantly reduce the level of MMP7 in serum or improve lung function compared to the corresponding changes in the control group [154].

Sodium cromoglycate is commonly used for treating allergic asthma, and studies have shown that tryptophan can reduce the activity of C-fiber sensory nerves by coupling with the orphan G protein-coupled receptor GPR35. PA101 is a new high-concentration sodium cromoglycate preparation that Birring et al. hypothesized might have the potential for treating coughs associated with IPF. They conducted a randomized double-blind phase-II clinical trial with 24 IPF patients who had a daytime objective cough frequency of ≥ 15 times/h. The patients were randomly assigned to receive inhaled PA101 (40 mg, three times/day) or a placebo using a PARI eFlow inhaler for 2 weeks. The results showed that the 24-h objective cough frequency in the inhaled PA101 group decreased significantly (by 31%) compared to that in the placebo group [155]. However, a phase IIB study in which PA101 was administered for treating IPF, conducted in 2022 showed that the difference between the two groups in improving cough was not significant [156].

Along with the above-mentioned studies, inhalation therapy with gene inhibitors has emerged as a treatment option for PF. The results of positron emission tomography scanning showed that inhaled GSK3008348 has the characteristics of rapid lung absorption, good targeting, and multiphasic metabolism in IPF patients [156]. A RCT of a Gal-3 inhibitor, TD139, for inhalation therapy based on interference with IPF showed that the concentration of TD139 in the lungs was more than 500 times higher than its concentration in the blood, and its half-life was up to 8 h. It not only significantly reduced the expression of Gal-3 in macrophages in the bronchoalveolar lavage fluid of the patients but also significantly reduced the plasma levels of pro-fibrotic factors such as PDGF and CCL-18. The inhalation of the dry powder of TD139 was safe and well-tolerated by IPF patients [157].

## Conclusions and future directions

In general, inhalation therapy is an effective treatment for PF. Although several challenges need to be overcome, such as low deposition rates in the lungs and biological barriers, inhalation therapy has significant advantages, such as direct targeting of the disease site, bypassing the first-pass effect of the liver, decrease in the severity of symptoms, and low dependence on other treatment strategies, treatment time, and cost.

With the continuous development of inhalation therapy, novel inhalation therapeutic techniques have made great progress in PF intervention, especially using MPs/NPs-mediated delivery, which has reached the preclinical stage. Antibiotics, anti-inflammatory/antioxidant drugs, TCM monomers, and other drugs are loaded into various forms of NPs and delivered to the lungs via nebulization or dry powder inhalation. These methods can overcome the barriers of low lung deposition and lung biological barriers, enhancing the progress of clinical treatment.

However, NPs as clinical diagnostic tools for PF have certain limitations, and whether the diagnosis and treatment of PF can be combined to achieve a unified diagnostic and treatment method for biopsy lesions is not clear. Inhalation therapy includes dry powder inhalation and nebulization inhalation, which have certain advantages, but their scope of application is relatively limited. Combining inhalation therapy with other technologies, such as phototherapy, radiation, or magnetism, might increase the efficacy of disease treatment. The number of clinical trials on inhaled therapies for treating PF is limited. Most studies have focused on basic pharmacology and the relief of symptoms. Thus, considerable research and progress are required before PF can be reversed or cured. Therefore, more comprehensive studies need to be conducted. The pathological and physiological mechanisms of PF need to be elucidated to address the root cause and investigate the potential of inhalation therapy for treating PF.

## Data Availability

Not applicable.
